# A game of tag: A review of protein tags for the successful detection, purification and fluorescence labelling of proteins expressed in microalgae

**DOI:** 10.1111/tpj.70272

**Published:** 2025-06-19

**Authors:** Jonathan Scarfe, Darius Kosmützky, R. Ellen R. Nisbet

**Affiliations:** ^1^ School of Biosciences University of Nottingham, Sutton Bonington Campus Loughborough LE12 5RD UK; ^2^ Department of Biochemistry University of Cambridge Cambridge CB2 1QW UK; ^3^ Present address: Sainsbury Laboratory University of Cambridge Cambridge CB2 1LR UK

## Abstract

Recombinant proteins play a crucial role in both fundamental research and biotechnology. In the laboratory, recombinant proteins are used in a myriad of ways, including to label cells, localize proteins and isolate complexes. In the clinic, antibody‐based therapeutics can dramatically increase cancer survival rates, while virus‐like particles (VLPs) are being developed as next‐generation vaccines. These innovations have escalated demands for biopharmaceutical recombinant proteins. However, in traditional systems (e.g. mammalian and microbial) the expression of recombinant proteins can be prohibitively expensive. One sustainable, low‐cost solution is to use a microalgal‐based expression system, such as *Chlamydomonas reinhardtii, Phaeodactylum tricornutum*, *Chlorella* sp., *Haematococcus pluvialis* or *Nannochloropsis gaditana*. Tools for microalgal protein expression are developing rapidly. Yet our understanding of recombinant protein expression and purification in microalgal systems lags that of traditional systems. Here, we review the impact of commonly used affinity and epitope tags (e.g. Polyhistidine‐tag, Strep‐tag II, HA‐tag and FLAG‐tag) on recombinant protein detection, purification and biofunctionality in microalgae. Additionally, we review fluorescent protein tags (such as GFP, mVenus, DsRed and mCherry) and protease cleavage sites, including ‘self‐cleaving’ 2A peptides. Finally, we provide guidance on experimental design to enhance the likelihood of successfully expressing recombinant proteins in microalgae.

## Significance Statement

Protein tags for detection, purification and fluorescence labelling are vital in microalgal research, yet the suitability of specific tags remains largely unexplored. This review discusses the pros and cons of a comprehensive list of protein tags and provides important guidance for experimental design within a microalgal context.

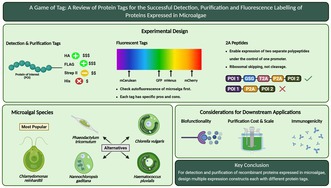

## INTRODUCTION

The use of recombinant proteins in research has revolutionized our study of the cell. Using fluorescent tags, we can localize individual proteins within a cell, while affinity tags give us the ability to carry out immunoprecipitation experiments, thus determining interaction partners. Overexpression of recombinant proteins allows us to determine protein structure and function via structural biology. Recombinant proteins also have a vast array of biotechnological applications from processing textiles (Kabir & Koh, 2022) to food production (Trono, 2019). They are also vital as therapeutics in treating and preventing cancer (Zhang et al., 2022), autoimmune disorders (An et al., 2023), genetic disorders (Yang & Montgomery, 2021) and infectious disease (Harper et al., 2004).

Traditional platforms for the expression of recombinant proteins include *E. coli*, yeast, mammalian cell lines, and insect cells (Tripathi & Shrivastava, 2019). Whilst these platforms have been successful in the production of various recombinant proteins, they are not without drawbacks. *E. coli* cells are prone to the production of inclusion bodies (insoluble aggregates composed mostly of proteins), which present a serious hurdle to overcome in the pursuit of obtaining soluble and biofunctional recombinant proteins (Bhatwa et al., 2021). Furthermore, post‐translational modifications in *E. coli* and yeast, such as glycosylation, can impair the biofunctionality of recombinant eukaryotic proteins expressed in these systems (Khow & Suntrarachun, 2012; Li et al., 2022). Mammalian cell platforms are notoriously expensive, especially on a commercial scale (Demain & Vaishnav, 2009), and possess the risk of viral contamination (Barone et al., 2020). Insect cells are less costly than mammalian cells, but they too are a relatively expensive platform to produce recombinant proteins (Tripathi & Shrivastava, 2019).

The production of recombinant proteins in eukaryotic microalgae is emerging as a possible alternative with several advantages that may circumvent some of the issues with traditional platforms (Banerjee & Ward, 2022; Dyo & Purton, 2018; Gong et al., 2011; Rasala & Mayfield, 2015). Firstly, phototrophic cultivation using inexpensive media minimizes costs and carbon footprint. The ‘Generally Regarded As Safe’ status attributed by the FDA to species such as *Chlamydomonas reinhardtii* (*C. reinhardtii*) and *Chlorella vulgaris* means that they do not share common pathogens with humans. Also, in contrast to *E. coli*, these microalgal species are safe to consume, which could be useful in the development of oral vaccines. Furthermore, the chloroplast organelle serves as a biosynthesis and storage compartment that also allows for precise transgene integration. Lastly, glycoengineering of the native glycosylation patterns in microalgae to mimic that of humans may be simpler to achieve than in other microbial platforms, as microalgae show more similar N‐glycosylation patterns to humans than *E. coli* or yeast (Dubey et al., 2023; Leprovost et al., 2024).

However, there are also limitations to microalgae as a platform for the expression of recombinant proteins. Clonal variability in transgene expression levels means that the screening of transformant colonies can be extremely important (Gutiérrez et al., 2022), although ultimately the levels of recombinant protein expression in *C. reinhardtii* are often reported to be relatively low in comparison to other expression platforms (Arias et al., 2023; Braun‐Galleani et al., 2015; Commault et al., 2020). This can present a serious challenge to researchers when downstream applications require high yields of recombinant protein, such as cryo‐electron microscopy.

Additionally, the use of protein tags in the expression of recombinant proteins in microalgae often needs to be optimized. Protein tags are short peptides that facilitate desired functions when attached to recombinant proteins. Such functions include increased solubility, detection, or purification of the recombinant protein. Protein tags play a pivotal role in the production of recombinant microalgal proteins. They can be the deciding factor in a recombinant protein's detection and purification (Derrien et al., 2012; Kiefer et al., 2022). Furthermore, a specific protein tag may permit expression, detection, and purification but hinder the bio‐functionality of the recombinant protein (Crozet et al., 2018), and the successful application of a tag in one study does not guarantee success with the same tag in other studies.

This review, summarised in Box 1, examines the advantages/disadvantages of various protein tags. Consequently, the conclusions made here aim to provide guidance on the choice of protein tag for future studies in the field of microalgal recombinant protein expression. Finally, research‐relevant open‐ended questions are posed in Box 2.

Box 1 **Summary bullet points**

- Experimental design should include the use of more than one protein tag to enhance the probability of successful protein expression, detection and purification.
- Expression, detection and purification of recombinant proteins is likely to be more successful using epitope tags (e.g. HA‐ and FLAG‐tag) rather than affinity tags (e.g. His‐tag and Strep‐tag II), though purification via epitope tags is vastly more expensive.
- Applications of fluorescent protein tags are highly diverse, and choice of tag must balance brightness, stability, maturation time and compatibility with the unique autofluorescent properties of microalgal cells.
- The use of self‐labelling enzyme tags in microalgal research is limited and their potential remains to be explored.
- The expression of multiple polypeptides driven by one promoter is currently problematic: the use of ‘self‐cleaving’ 2A peptides show varying degrees of success and may not work in chloroplasts.

Box 2 **Open‐ended Questions**

- Can we design strains of *Chlamydomonas reinhardtii* to reduce the co‐purification of endogenous protein during protein isolation, similar to what has been achieved in *E. coli* for Ni^2+^‐NTA IMAC?
- Is *Chlamydomonas reinhardtii* truly the best microalgal species for recombinant protein expression, or should another model species be chosen?
- Do alternate microalgal species display endogenous protein co‐purification as is seen in *Chlamydomonas reinhardtii*?
- Is the broad spectrum of fluorescent proteins functional and accessible for studies in non‐model microalgal species?
- Can self‐labelling enzyme tags (e.g. SNAP, Halo tag) significantly contribute to field of microalgal research?

## PROTEIN TAGS USED IN MICROALGAE RECOMBINANT PROTEIN EXPRESSION

The most commonly used protein tags in microalgae are haemagglutinin (HA)‐, FLAG‐, Streptavidin (Strep) II‐, and Polyhistidine (His)‐tags. Table 1 summarizes the general features, advantages and disadvantages, and reported successes or failures of each tag, specifically when employed within a microalgal setting. See Tables S1–S4 for more detailed tables on each protein tag.

**Table 1:** Summary of commonly used protein tags for the detection and purification of microalgae‐expressed recombinant proteins, including respective advantages and disadvantages

Protein tag	Description	Advantages	Disadvantages	Rating
HA	Epitope tag derived from human influenza and comprised of the amino acids YPYDVPDYA.	Many successful applications within *C.reinhardtii*. Has succeeded where the His‐tag and Strep‐tag II have failed. Highly specific.	Very expensive for purification. Purification is not always successful with *C.reinhardtii*.	A
FLAG	Artificial epitope tag with the amino acid sequence DYKDDDDK.	Apparent absence of issues when used in microalgae, although there are fewer reports than the HA‐tag. Built‐in cleavage site.	Very expensive for purification. Low binding capacity. Tag cleavage is often required post‐purification.	A
Strep‐tag II	Affinity tag comprised of the amino acids WSHPQFEK.	Has succeeded where the His‐tag has failed. Good level of specificity. Less expensive than epitope tags for purification.	Can impact biofunctionality. More expensive than other affinity tags with lower binding capacities.	B
His‐tag	Peptide affinity tags are typically comprised of 3–10 consecutive histidine residues.	Has been successfully employed in various microalgae species. Relatively cheap purification. Suitable for native and denaturing conditions.	Co‐purification of endogenous *C.reinhardtii* proteins. Can impact biofunctionality and yield.	C

Note: The rating column, with ‘A’ being the best rating and ‘C’ being the worst, signifies the respective tag's level of performance within microalgae based on evidence within the literature.

Various experimental considerations affect the expression of recombinant proteins independent of the identity of the tag. The positioning of the tag can be crucial when native transit peptides need to be maintained. For example, tagging at the C‐terminus is preferred if the protein has an N‐terminal localization signal or vice versa. It may also be necessary to test both termini if the effect of tagging on protein function is unclear. As such, the protein tag position (N‐ or C‐terminal, has been recorded for each study within the Tables S1–S4. However, there are no observable patterns from the studies discussed to suggest a favourable tag position or transformation location for any of the protein tags.

There are multiple different cloning strategies available to the researcher that enable the addition of protein tags to genes of interest (GOIs) and the subsequent insertion of GOIs into expression vectors. These strategies include the Golden Gate‐derived MoClo toolkit for nuclear transformation (Crozet et al., 2018), the MoCloro toolkit for chloroplast transformation (Melero‐Cobo et al., 2025), and recombineering for the cloning of complex genes without relying on polymerase chain reaction (Emrich‐Mills et al., 2021). Furthermore, there are several ways in which to genetically transform microalgae, with glass bead agitation, micro‐particle bombardment and electroporation being commonly used methods (Sreenikethanam et al., 2022).

### HA‐tag

The HA‐tag (YPYDVPDYA) is an epitope tag composed of amino acids 98–106 of the human influenza virus HA protein (Young et al., 2012). It shows strong immunoreactivity, which makes HA‐tagged proteins easily detectable with commercially available anti‐HA monoclonal antibodies (mAbs) (Zhao et al., 2013). Purification of HA‐tagged recombinant proteins is also possible by using anti‐HA mAbs that are covalently immobilized on a resin such as agarose (DeCaprio & Kohl, 2020). The distinct advantage of the HA epitope tag is its specificity: the use of mAbs for purification makes the process highly specific (DeCaprio & Kohl, 2020). However, due to the inherent immunogenicity of the HA tag, cleavage of the tag post‐purification is often needed to remove any impact on downstream analyses such as immunization trials. This requires a cleavage site to be placed between the protein of interest (POI) and the HA‐tag. Such protein‐tag cleavage, which can be challenging and tedious, is not necessarily required for less immunogenic tags. Another drawback of HA‐tag systems is that the use of mAbs for purification is very expensive in comparison to direct purification. Gentle elution of HA‐tagged proteins from mAb resins is possible using commercially available HA synthetic peptide, or they can be eluted using acidic glycine, sodium thiocyanate or sodium hydroxide solutions (Zhao et al., 2013).

HA‐tags have been highly successful in *C. reinhardtii* (Table S1). Crozet et al. (2018) employed the rapamycin‐insensitive *rap2 (∆FKBP12)* mutant of *C. reinhardtii* to investigate the suitability of various protein fusion tags for the reintroduction and detection of FKBP12 to the microalgae, which should confer rapamycin sensitivity. Sensitivity to rapamycin was obtained with an HA‐tag fused to FKBP12. Other studies have also employed the HA‐tag for detection purposes but did not purify the POI (Larrea‐Alvarez & Purton, 2020) or rely on a different purification method than the HA‐tag (Ide et al., 2016; Liu et al., 2020). There have been successful purifications of recombinant proteins from microalgae using HA mAb resins: flagella proteins (Lechtreck et al., 2009), ribosomal proteins (Westrich et al., 2021) and photosystem I assembly factors (Nellaepalli et al., 2018). In each study, the proteins expressed were correctly folded and helped to elucidate their respective biological functions, underlining the usefulness of the HA‐tag for functional characterizations.

There is a paucity of reported issues with HA resin purification of microalgal‐expressed recombinant proteins. This could either be because expression of proteins with an HA‐tag is generally successful, or it could be that unsuccessful expression attempts have not been published. Only one study was found reporting unsuccessful purification of HA‐tagged SARS‐CoV‐2 spike protein expressed in *C. reinhardtii*, despite successfully detecting the protein by western blot using an anti‐HA antibody (Kiefer et al., 2022). A number of underlying reasons were suggested: incorrect folding of the tag, proteolytic truncation of the tag, or modifications to the protein as it passed through the secretory pathway (Kiefer et al., 2022).

### FLAG‐tag

The FLAG‐tag (DYKDDDDK) is an artificial hydrophilic epitope tag (Young et al., 2012). Like the HA tag, the FLAG epitope tag is immunogenic and readily detectable and purifiable via mAbs. The M1, M2, and M5 mAbs have been developed for the detection and immunoprecipitation of FLAG‐tagged recombinant proteins (Zhao et al., 2013), each of which varies in recognition site and enables different binding conditions. FLAG‐tag systems are hindered by the price of purification resins, their relatively low‐binding capacities (Yadav et al., 2016) and that they may also need to be cleaved from the POI post‐purification. However, the unique advantage of the FLAG tag is that its sequence contains an enterokinase cleavage site (DDDDK), meaning a separate cleavage site is not required between the tag and the fusion protein (Young et al., 2012). FLAG‐tagged proteins can be eluted from resins by either an acidic glycine buffer or by commercially available FLAG peptides.

FLAG‐tag use in *C. reinhardtii* has generally been successful (Table S2). Detection of the FLAG‐tagged FKBP12 protein analysed by Crozet et al. (2018) was achieved by western blot using an anti‐FLAG antibody, and the POI was fully functional. Immunoprecipitation of FLAG‐tagged recombinant proteins expressed in microalgae has also been achieved (Demurtas et al., 2013; Jones et al., 2013; Rasala & Mayfield, 2015; Torres‐Tiji et al., 2022). Jones et al. (2013) employed anti‐FLAG mAb resin to purify the malaria vaccine candidate Pfs48/45 from *C. reinhardtii*, with subsequent analysis indicating the protein possessed the correct conformation for protective effects; although it was noted that further analysis was required to confirm the protein's biofunctionality. Rasala et al. (2010) detected, purified and confirmed the biofunctionality of two therapeutic human proteins tagged with FLAG: human vascular endothelial growth factor (hVEGF) isoform 121 and high mobility group protein B1 (HMGB1). Using standard bioactivity assays, VEGF and HMGB1 expressed in *C. reinhardtii* were found to have a similar activity to the same proteins from bacterial expression systems (the platform traditionally used to express these proteins). Furthermore, FLAG‐tagged human papillomavirus (HPV) 16 E7GGG was expressed in *C.reinhardtii* at 6x higher levels than its His‐tagged counterpart (see Section 2.4) in Demurtas et al. (2013) whilst also affording cancer protection in an animal model, demonstrating the protein's biofunctionality. The lower expression levels observed with the His‐tagged variant were suggested to be due to the impairment of an unspecified chloroplast function by the His‐tag peptide, which resulted in significant inhibition of cell growth (Demurtas et al., 2013). Torres‐Tiji et al. (2022) fused the medically relevant human intercellular adhesion molecule‐1 (ICAM‐1) (Van De Stolpe & Van Der Saag, 1996) to a FLAG tag and successfully detected and purified the protein, resulting in very high concentrations of ICAM‐1 (46.6 mg/L). However, it is important to note that media concentrations were optimized and anion exchange chromatography (AEC) was conducted prior to immunoprecipitation with an anti‐FLAG resin. Nonetheless, the recombinant ICAM‐1 was observed to perform at a similar level to mammalian cell‐expressed ICAM‐1 in native ligand binding assays.

It is not apparent within published research that there exist problems with the expression, detection, or purification of FLAG‐tagged recombinant proteins in *C. reinhardtii*. However, the high price of anti‐FLAG resins makes the scaling up of FLAG‐tagged protein expression and purification extremely expensive, which may make the system impractical for the large‐scale production of proteins in microalgae.

### Strep‐tag

The Strep‐tag was originally a nine‐amino‐acid peptide (AWRHPQFGG) that displayed a very high intrinsic affinity for the protein streptavidin (Korndörfer & Skerra, 2002). The Strep‐tag and streptavidin have both been subjects of engineering to optimize their binding affinity, resulting in the Strep‐tag II (WSHPQFEK) and Strep‐Tactin, respectively (Kimple et al., 2013; Maertens et al., 2015). Strep‐Tactin can be conjugated to matrices to allow affinity chromatography of proteins fused to a Strep‐tag II. Strep‐tags bind specifically to Strep‐Tactin, covering the Strep‐Tactin binding sites of the natural ligand biotin (Maertens et al., 2015). Biotin is used to elute Strep‐tagged proteins (Kimple et al., 2013). The downsides of the Strep‐tag II system are the moderately expensive price of its purification resins, in addition to their relatively lower binding capacity compared to IMAC columns (Section 2.4) (Maertens et al., 2015; Zhao et al., 2013). On the other hand, in comparison to other affinity tags, eluates of a Strep‐tag II system are often of higher purity due to the highly specific binding, and the system is also applicable for pH‐pH‐sensitive proteins (Schmidt & Skerra, 2007).

Strep‐tags have been successfully used in *C. reinhardtii* (Table S3). By switching from a His‐tag to a Strep‐tag II system, Derrien et al. (2012) were able to detect and purify the chloroplast Clp peptidase (ClpP) complex of *C. reinhardtii*. Not only did the Strep‐tag II yield much less co‐purification of endogenous proteins than observed with the His‐tag, but it also resulted in a much better yield than a tandem affinity purification (TAP)‐tagged counterpart. The Strep‐tag II system yielded ClpP at a concentration of 80 μg/L of culture, whereas the TAP‐tag resulted in <30 μg/20 L of culture. Similar results were achieved by Pivato et al. (2021), where the photoactive orange carotenoid‐binding protein 2 (OCP2) was Strep‐tagged and purified from *C. reinhardtii* using a Strep‐tactin affinity resin. These studies also report on further successful analyses such as tandem mass spectroscopy (Derrien et al., 2012) and tests on functionality (Pivato et al., 2021), demonstrating that recombinant proteins fused with the Strep‐tag II can result in correctly folded proteins of proper function.

However, the use of a Strep‐tag II does not guarantee biofunctionality or sufficient protein expression levels. Crozet et al. (2018) were able to detect Strep‐tagged FKBP12 protein via western blot using both protein‐specific and anti‐Strep‐tag II antibodies, but the Strep‐tagged FKBP12 was functionally impaired, resulting in a rapamycin‐insensitive phenotype. Furthermore, Jarquín‐Cordero et al. (2020) observed expression levels of Strep‐tagged human hVEGF that were extremely low. It should be noted, however, that the low expression levels in this study may be due to differences in expression vectors and not because of an impact that the Strep‐tag II had on the recombinant protein.

### His‐tag

His‐tags are peptide affinity tags typically comprised of 3–10 consecutive histidine residues (e.g. HHHHHH for the 6x His‐tag) fused to a POI (Young et al., 2012). Immobilised metal‐affinity chromatography (IMAC) takes advantage of the covalent bonds that form between histidine residues and transition metals (Ni^2+^, Co^2+^, Cu^2+^, and Zn^2+^) to purify His‐tagged proteins from crude protein extracts; no other amino acid exhibits a stronger affinity with transition metals (Bornhorst & Falke, 2000). Ni^2+^‐nitriloacetic acid (Ni^2+^‐NTA) agarose resin is a commonly used metal matrix in IMAC. The compound imidazole is used to elute bound His‐tagged proteins from the IMAC matrix by competitive displacement, theoretically resulting in a pure sample of the POI (Young et al., 2012). His‐tags are often used because the resins are widely available and relatively inexpensive but still offer good yields of purified recombinant proteins whilst also being suitable for denaturing and native conditions (Lichty et al., 2005). However, His‐tags are notorious for the co‐purification of endogenous proteins from some expression platforms such as *E. coli* (Andersen et al., 2013) and *C. reinhardtii* (Derrien et al., 2012), which is also echoed by our experiences (unpubl. Data). Lastly, His‐tags are not suitable for proteins that prefer a low pH, as such conditions impair the ability of histidine to interact with the metal – lowering the pH is an alternative method to elution when imidazole is not appropriate (Bornhorst & Falke, 2000).

His‐tags have been successfully employed for the purification of recombinant proteins expressed in microalgae (Table S4). His‐tagged recombinant human erythropoietin and the white spot syndrome virus (WSSV) envelope protein VP‐28 were expressed in *C. reinhardtii* by Eichler‐Stahlberg et al. (2009) and Kiataramgul et al. (2020), respectively. The proteins were purified via Ni^2+^‐NTA IMAC, and their identities were confirmed by western blot. *C. reinhardtii* has also allowed the expression of a His‐tagged protein where more traditional platforms have failed. Meng et al. (2016) reported difficulties in expressing biofunctional derivatives of the antimicrobial peptide mytichitin‐A in *E. coli* and yeast. In a later study, switching to *C. reinhardtii* enabled the expression of His‐tagged mytichitin‐A with superior biofunctionality (Dong et al., 2018). The *C. reinhardtii* expressed mytichitin‐A, which was detected by Coomassie blue staining after Ni^2+^‐NTA affinity purification and size exclusion chromatography.

In contrast, there are reports of His‐tags causing a variety of issues for the expression of recombinant proteins in microalgae. Derrien et al. (2012) anecdotally reported that they observed large levels of proteins distinct in size from the POI co‐purifying when using Ni^2+^‐NTA resin to purify His‐tagged proteins from *C. reinhardtii*. They conducted a BLAST search of six consecutive histidine residues that revealed hundreds of endogenous *C. reinhardtii* proteins contained polyhistidine stretches, indicating that the large amount of contamination likely originated from endogenous proteins (Derrien et al., 2012). Kiefer et al. (2022) also unsuccessfully employed Ni^2+^‐NTA IMAC, with *C. reinhardtii‐*expressed SARS‐CoV‐2 spike protein remaining in the flow‐through fraction. Furthermore, it has been observed that His‐tags can impact the biofunctionality of proteins. Expression of His‐tagged FKBP12 by Crozet et al. (2018) resulted in a phenotype that remained insensitive to rapamycin, therefore indicating improper functionality. The His‐tag has also been shown to impact protein yield. Demurtas et al. (2013) successfully expressed, Ni^2+^‐NTA purified and detected His‐tagged HPV 16 E7 protein produced in the chloroplast of *C. reinhardtii*, but it was expressed at lower levels compared to its FLAG‐tagged counterpart (see Section 2.2).

### Alternative tags

The protein tags discussed so far are the most commonly used tags for the detection and purification of microalgal‐expressed recombinant proteins. However, there have been other tags employed for such purposes, but on a less frequent basis.

The V5 epitope tag (GKPIPNPLLGLDST) derives from the Pk epitope found on the P and V proteins of the simian virus 5 (Hanke & Randall, 1995). The V5‐tag has been used successfully to detect *C. reinhardtii‐*expressed recombinant proteins via western blotting with anti‐V5 primary antibodies (Gutiérrez et al., 2022; Kong et al., 2017; Kong et al., 2018). Although it is notable that purification via the V5 tag was not attempted in any of those studies.

The Myc‐tag (EQKLISEEDL), another epitope tag, is derived from the c‐Myc oncoprotein (Evan et al., 1985). This protein tag has been used to successfully detect microalgal‐expressed recombinant protein via western blotting using an anti‐Myc antibody (Crozet et al., 2018; Gutiérrez et al., 2022), but as with the V5 tag, purification was not attempted.

Lastly, Gutiérrez et al. (2022) employed an E‐tag (GAPVPYPDPLEPR), another epitope tag, to successfully detect *C. reinhardtii‐*expressed mVenus via western blotting using an anti‐E‐tag antibody; no purification was attempted.

## ALTERNATIVE MICROALGAE SPECIES

There are various strains of *C. reinhardtii* to choose from depending on scientific objective and experimental design, e.g. TN72 (Wannathong et al., 2016) for chloroplast expression using photosynthetic selection or UVM4 for nuclear expression using antibiotic selection (Neupert et al., 2009). Moreover, the discussion thus far has been inadvertently centred on *C. reinhardtii* due to its overwhelming popularity in studies aiming to express recombinant proteins using microalgae. However, there are also alternate species of microalgae to *C. reinhardtii* that have been used to express recombinant proteins. Unfortunately, only a few of them also employ a protein tag for detection or purification purposes. Nonetheless, it is important to acknowledge that recombinant protein detection and purification are possible in alternative microalgal species.

The His‐ and FLAG‐tags are commonly used protein tags in a range of alternative microalgal species. Slattery et al. (2022) used an anti‐His antibody to detect *Phaeodactylum tricornutum* expressed SARS‐CoV‐2 receptor‐binding domain (RBD) and then purified the POI via an Ni^2+^ IMAC resin. Interestingly, binding issues were reported between the His‐tagged RBD and the Ni^2+^ IMAC resin, although these problems were attributed to a tobacco etch virus (TEV) protease site fused between the His‐tag and POI. The His‐tag has also been successfully used for both detection and purification of recombinant protein expressed in *Chlorella* sp. (He et al., 2018), and solely for detection purposes in *Haematococcus pluvialis* (Wang et al., 2020) and *Nannochloropsis gaditana* (Cui et al., 2021). Positive results using the FLAG tag for detection via western blotting with an anti‐FLAG antibody have been observed in *Phaeodactylum tricornutum* (Hao et al., 2018), *Chlorella* sp. (Lee et al., 2020), *Nannochloropsis gaditana* (Lin et al., 2024) and Haematococcus *pluvialis* (Galarza et al., 2018).

Other protein tags than the His‐ or FLAG‐tag for the detection and purification of recombinant proteins expressed in alternative microalgal species have only been reported scarcely. For example, Zhang et al. (2019) used the V5‐tag to successfully detect *Phaeodactylum tricornutum* expressed FuT54599, a protein involved in fucosylation, but further usage of the V5‐tag in other microalgal species is not apparent within the literature. The theme of sparsity is continued when searching for the use of other tags in alternative microalgal species. Researchers interested in non‐model microalgae species, therefore, best rely on comparable results from *C. reinhardtii* to make informed decisions.

## FLUORESCENT PROTEIN TAGS IN MICROALGAE

For the fundamental researcher, many of the above tags can also be useful for investigating the localization of a protein within the microalgal cell, e.g. via immunofluorescence microscopy. Although, more common for localization studies are fluorescent protein tags, which can also be useful for other applications. This section reviews the key applications of fluorescent tags, considerations for their use in microalgal systems, and the types available, with a focus on *C. reinhardtii* as a model organism.

### Applications of fluorescent proteins in microalgal research

Fluorescent proteins (FPs) have a wide range of uses in microalgae, including for proof of genetic tractability, transformant screening, expression level monitoring, localization studies, protein–protein interaction (PPI) assays, and even protein purification.

Recent examples of microalgae that were shown to be genetically tractable via the expression of FPs include *Tetradesmus* (Guo et al., 2013; Muñoz et al., 2019), *Cyanidioschyzon* (Fujiwara et al., 2015), *Neochloris* (Muñoz et al., 2019), and many more (Faktorová et al., 2020) (Table S5).

Further, FPs have become essential tools for screening transgenic microalgal transformants and monitoring protein expression levels, particularly in applications where rapid identification of high‐expression strains is needed (Table S6). Fluorescence intensity directly correlates with the amount of FP and, by extension, the expression level of the fused target protein. Especially in *C. reinhardtii*, this has facilitated synthetic biology and metabolic engineering advances for the expression of heterologous proteins (Einhaus et al., 2021; Lauersen et al., 2018; Mehrshahi et al., 2020). Alternative to FPs, bioluminescent proteins such as luciferase and its redesigned version NanoLuc can be used as tags for the estimation of expression levels (Crozet et al., 2018).

Confocal microscopy of FP‐tagged proteins has allowed detailed mapping of protein localization within microalgal cells (Table S7), with various FPs shown to be functional in specific organelles such as the mitochondria, chloroplast, and nucleus in *C. reinhardtii* (Crozet et al., 2018). Also in *C. reinhardtii*, Wang et al. (2023) used a Venus fusion to map over 1000 proteins in the chloroplast, providing valuable insights into chloroplast function and protein compartmentalization.

Techniques like bimolecular fluorescence complementation (BiFC) and Förster resonance energy transfer (FRET)‐based interaction studies are popular tools for the study of PPIs but are not widely used in microalgae. In BiFC, two proteins of interest are tagged with split fragments of an FP. Fluorescence is only observed when the proteins are in close proximity, bringing the FP fragments together (Xing et al., 2016). This technique, however, has a high rate of false positives, requiring rigorous experimental controls (Horstman et al., 2014). A recent study demonstrated BiFC in *C. reinhardtii* using split‐YFP via transient expression from introduced RNA (Ye et al., 2024). FRET‐based approaches rely on putative interacting proteins being labelled with a different FP, such as Cerulean and Venus, which form an efficient FRET pair (Rasala et al., 2013). While its use for PPI investigation in microalgae remains limited, the FRET‐based calcium sensor Yellow Cameleon, which is reliant on CFP and YFP, has been shown to be functional in different cellular compartments of *C. reinhardtii* (Pivato et al., 2023, 2025). In addition to the fluorescent characteristics of FPs, they also represent popular targets for protein purification. For example, relying on the binding of immobilized antibodies, interaction partners of a GFP‐tagged immunophilin from *C. reinhardtii* were discovered (Fu et al., 2023). However, antibody‐based methods often come with significant costs. As an inexpensive and easily accessible alternative, designed ankyrin repeat proteins (DARPins) that specifically bind GFP were developed (Hansen et al., 2017).

### Types of fluorescent tags

There are four primary methods for fluorescently labelling proteins. The most common approach involves fusing a protein of interest to an FP derived from naturally occurring fluorescent proteins, such as green fluorescent protein (GFP) from the jellyfish *Aequorea victoria*. Other methods include self‐labelling enzyme tags like Halo and SNAP/CLIP tags, PRIME labelling, and biarsenical dyes (Crivat & Taraska, 2012). While these alternatives offer unique advantages, they have not been widely applied in microalgae, possibly due to technical challenges or lack of established protocols. Here, we focus on the first two approaches, with an emphasis on GFP‐derived fluorescent proteins, which have proven highly beneficial for microalgal research.

### Fluorescent protein tags for microalgal studies

A wide range of fluorescent proteins is available to researchers, with variants spanning the visible spectrum from blue to red (see FPbase.com (Lambert, 2019)). The most used FPs in microalgae are GFP derivatives like Venus, mVenus, and mCerulean and DsRed derivatives such as mCherry. The lowercase ‘m’ at the start of the name of some FPs stands for ‘monomeric’ and indicates that they have been modified to prevent the formation of dimers. All these proteins are structurally similar to GFP and typically consist of around 240 amino acids (~27 kDa) arranged in an 11‐stranded beta‐barrel structure that encloses the chromophore (Chudakov et al., 2010). They generally have little impact on target protein function, can be fused to either the N‐ or C‐terminus, and mature quickly. The maturation process includes the folding of the FP as well as the catalytic reaction with oxygen that results in the formation of the chromophore. The chromophore is greatly influenced by the amino acid environment of the FP, which has been harnessed to create differently coloured FP variants.

When selecting an FP for microalgal studies, several considerations come into play.

First, choosing an FP with the appropriate excitation and emission spectra is crucial, as the autofluorescent pigments in microalgae (such as chlorophylls and carotenoids) can interfere with detection. In *C. reinhardtii*, FPs across the blue to red spectrum have been successfully used, including mTagBFP, mCerulean(3), mTFP1, VFP, GFP, Clover, (m)Venus, YFP, LSSmOrange, tdTomato, mScarlet, mRuby2, LSSmCherry, and mCherry (Table S6). However, blue and green FPs such as mTagBFP, mCerulean, and GFP can have higher background signals due to autofluorescence, while red‐shifted FPs like Venus, mCherry, or tdTomato generally have a higher signal‐to‐noise ratio (Rasala et al., 2013).

Second, FP stability and pH sensitivity need to be considered. Red‐shifted FPs such as tdTomato or mCherry are derived from DsRed rather than GFP, and greater incidences of misfolding have been reported in organisms other than microalgae (Crivat & Taraska, 2012). Some FPs, including EGFP, Clover, Venus, and YFP, are more sensitive to acidic environments, as indicated by their higher pK_a_ value (above 6) (Shaner et al., 2005). This is especially relevant for target proteins associated with photosynthesis and/or a thylakoid lumenal localization as the lumen acidifies under illumination. In fact, a large‐scale study of chloroplast protein localization in *C. reinhardtii* could localize 1034 out of 3117 proteins tagged with C‐terminal Venus. While this underlines how versatile Venus is, two thirds of successfully cloned proteins could not be localized. The authors suggested that low expression levels may have led to this limitation (Wang et al., 2023). However, the acid sensitivity of Venus could have potentially contributed to some unsuccessful localization efforts. Nevertheless, some luminal proteins were localized using Venus in this study, e.g. the electron carrier plastocyanin or the photosystem II assembly factor HCF136 (Wang et al., 2023).

Third, the importance of tag positioning (see Section 2) and linker requirements should be considered. Adding a flexible linker containing glycine and serine residues (e.g. ‘GSGSGS’) between the tag and the protein can improve the folding of the fusion protein and is usually good practice (Mehrshahi et al., 2020; Perozeni et al., 2020).

### Self‐labelling enzyme tags as alternatives to FPs

While FPs are powerful tools, they have some limitations. They are not very bright compared to organic dyes, can show blinking, and can be limited in their photostability and overall fold. Self‐labelling enzyme tags like Halo and SNAP/CLIP tags offer an alternative as they rely on a different protein fold and covalently attach to externally added fluorescent organic dyes *in vivo*. The organic dyes are thus independent of the protein tag and can assume highly customized spectral characteristics. These tags are similar in size to FPs and can also be placed at the N‐ or C‐terminus of the target protein.

SNAP and CLIP tags are engineered alkylguanine‐DNA‐alkyltransferases from humans and can attach to O^6^‐alkylguanine or O^6^‐benzylcytosine substrates, respectively. The former has been used to study flagellar proteins and their localization in *C. reinhardtii* (Fu et al., 2024; Gui et al., 2019). The Halo tag originates from a bacterial haloalkane dehydrogenase and catalyses the covalent attachment of a fluorophore to the active site aspartate via a haloalkane linker (Los et al., 2008). Its application for tagging proteins in microalgae remains to be tested.

## PROTEASE CLEAVAGE SITES

### Standard protease sites

Protease cleavage is an irreversible post‐translational modification (PTM) in which the peptide bond between specific amino acids is broken using protease enzymes, resulting in two distinct polypeptides (Li et al., 2020). Protease cleavage sites are the unique sequences of amino acids that specific enzymes will recognize and within which the cleavage will occur (Li et al., 2023). For example, the TEV protease recognizes the amino acid sequence ENLYFQ↓S, with ‘↓’ indicating where the cleavage occurs.

It is important to consider protease cleavage sites when considering protein tags as often a cleavage site is required between the protein tag and POI so that after protein detection and purification the tag can be removed. For example, not only are epitope tags often removed due to their inherent immunogenicity (Young et al., 2012), but various other protein tags are also cleaved due to their impact on POI crystallization (Bucher et al., 2002) and binding properties (Goel et al., 2000).

However, the enzymatic cleavage of protein tags from recombinant protein expressed in microalgae is rarely mentioned within the literature. Therefore, it is impossible to provide meaningful comments on the reliability of individual proteases when used in this context.

### 2A peptides

Viral‐derived 2A peptides enable the expression of multiple polypeptides under the control of a single promoter region (Z. Liu et al., 2017). Such ‘self‐cleaving’ 2A peptides are placed between open reading frames (ORFs), allowing expression of two distinct proteins. Despite the name, the sequence is not cleaved, but instead the expression of distinct proteins is obtained via the process of ribosomal skipping (Donnelly et al., 2001). In contrast to standard protease sites, the use of 2A peptides in microalgae is commonly discussed. The studies summarized in Table S8 provide examples of how the use of 2A peptides in microalgae has resulted in varying degrees of success.

Success was seen by Rasala et al. (2012) in which GFP and the antibiotic resistance marker *ShBle* were fused together using the foot‐and‐mouth‐disease‐virus 2A (F2A) peptide and expressed in the *C. reinhardtii* cytosol. The processing efficiency (i.e. the percentage of the construct that was expressed as individual polypeptides, rather than remaining fused together) of the 2A peptide was not calculated, but it was reported that the majority of the F2A peptide was processed and only a small amount of protein in the cytosol remained fused (Rasala et al., 2012). The processing efficiency of 2A peptides was reported by Dehghani et al. (2020), in which a novel chimeric 2A peptide comprised of a glycine‐serine‐glycine spacer, Thosea asigna virus (T2A) and Porcine teschovirus‐1 (P2A) (i.e. GSG‐T2A‐P2A) was used to fuse human interleukin‐2 to chloramphenicol acetyltransferase. Expression was analysed in *C. reinhardtii*, *Chlorella vulgaris* and *Dunaliella salina*, with no proteins remaining fused in the cytosol of each species. Koh et al. (2018) found success with *Nannochloropsis salina* in their analysis of *sfCherry* and *ShBle* sequences fused together by four separate 2A peptides: T2A, equine rhinitis A virus 2A (E2A), P2A and F2A. Respectively, they found average processing efficiency rates of 40%, 37%, 30% and 21%. However, Gornik et al. (2022) attempted to express GFP and puromycin N‐acetyltransferase (*pac*) in the dinoflagellate *Breviolum minutum* from the same promoter by employing the P2A peptide and they found the P2A peptide to be highly unreliable.

## CONCLUSIONS, GUIDANCE AND OUTLOOK

In recent years, protein tags have been established as indispensable tools for studying proteins in microalgae, particularly *C. reinhardtii*. Affinity and epitope protein tags have been employed for protein detection and purification, whereas fluorescent protein tags have enabled the study of protein localization and interaction. This review aims to serve as a guide on the most commonly used protein tags within the field of microalgal protein expression, aiding informed choices of protein tags during experimental design.

Epitope tags such as the HA‐tag or FLAG‐tag appear to be less problematic compared to affinity tags like the His‐tag or Strep‐tag II. However, it is important to note that whilst there are few reports of problems with epitope tags in microalgae, there may exist notable issues that have not been reported. Nonetheless, it is advisable that when designing studies of recombinant protein expression in microalgae to employ more than one protein tag for detection and purification purposes. For example, by creating both His‐tagged and FLAG‐tagged transformant strains, Ni^2+^‐NTA purification via the His‐tag can be attempted first, whilst, in the case of failure, reserving the potentially less problematic but more expensive method of immunoprecipitation via the FLAG‐tag. For His‐tags particularly, a key issue is the reliability of Ni^2+^‐NTA purification from *C. reinhardtii*. As such, it would be prudent to engineer new strains of *C. reinhardtii* that do not produce as much background co‐purification of endogenous proteins, as has been achieved in *E. coli* (Andersen et al., 2013).

Compared to affinity and epitope tags, fewer issues have been reported on the use of FPs in microalgae. The diversity of available FPs allows researchers to choose tags that balance brightness, stability, maturation time, and compatibility with the unique autofluorescent properties of microalgal cells. Meanwhile, self‐labelling enzyme tags such as SNAP and Halo tags are emerging as promising alternatives, providing additional flexibility in spectral features. As genetic engineering in microalgae continues to expand, further development and testing of these tags, particularly in less explored species, will be essential. Advances in tag technology, such as more photostable or more acid‐resistant variants, will also improve the accuracy and reliability of localization and expression studies. The continuous refinement of fluorescent protein and enzyme‐based tags will enhance our ability to probe the cellular functions of proteins in microalgae, contributing to broader applications in bioengineering, synthetic biology, and metabolic engineering.

The expression of recombinant proteins in alternative microalgal species to *C. reinhardtii* is scarcely reported. Nonetheless, the same principles discussed within this review surrounding *C. reinhardtii* are likely to apply to other species. In the future, with researchers conducting more studies on the use of alternate microalgal species for such purposes, the specific interactions that each species has with different tags will hopefully become apparent.

Finally, it is evident that protease cleavage sites receive little mention in studies aiming to express recombinant proteins in microalgae. It may be worthwhile investigating the activity of a wide range of proteases in cleaving protein tags from microalgal‐expressed recombinant proteins to determine if there is a more favourable enzyme for this context. When considering the use of 2A peptides to express multiple peptides from one promoter, it is advisable to once again employ more than one option, with the chimeric GSG‐T2A‐P2A being included in the selection.

There are great advantages to expressing recombinant proteins in microalgae compared to traditional platforms such as bacteria, mammalian cells, and yeast . Research using traditional platforms is expedited by the many useful resources and guides that have been developed over many years to guide experimental design and function. To access the full range of benefits that microalgae offer in recombinant protein expression, we need to develop microalgal‐specific resources. We hope that this guide will assist researchers in the rapidly expanding field of microalgal biotechnology, both during experimental design and during troubleshooting, allowing the successful production of recombinant proteins in microalgae.

## Author Contributions

All authors designed the research, performed the research, analysed the data, and wrote the paper.

## Supporting information


**Table S1.** Summaries of the relevant findings from studies that have employed a HA‐tag for either the detection or purification of recombinant proteins expressed in the green microalgae *Chlamydomonas reinhardtii*.
**Table S2.** Summary of studies that have employed a FLAG tag for either the detection or purification of recombinant proteins expressed in the green microalgae *Chlamydomonas reinhardtii*.
**Table S3.** Summaries of the relevant findings from studies that have employed a Strep‐tag for either the detection or purification of recombinant proteins expressed in the green microalgae *Chlamydomonas reinhardtii*.
**Table S4.** Summaries of the relevant findings from studies that have employed a His‐tag for either the detection or purification of recombinant proteins expressed in the green microalgae *Chlamydomonas reinhardtii*.
**Table S5.** Summary of studies that have employed FPs for proving the genetic tractability of a microalgal species.
**Table S6.** Summary of studies that have employed FPs for screening of expression levels mostly in the green microalga *Chlamydomonas reinhardtii*.
**Table S7.** Summary of studies that have employed FPs for localisation studies mostly in the green microalga *Chlamydomonas reinhardtii*.
**Table S8.** Summaries of studies that have employed ‘self‐cleaving’ 2A peptides in the expression of recombinant proteins in various microalgae species.

Supplementary Tables References

## Data Availability

Data sharing is not applicable to this article as no new data were created or analyzed in this study.
