# Supplementary material for: A game of tag: A review of protein tags for the successful detection, purification and fluorescence labelling of proteins expressed in microalgae
Source: Plant J. 2025 Jun 19;122(6):e70272. doi: 10.1111/tpj.70272 (PMC12178573; doi:10.1111/tpj.70272)
Supplement: Supplementary file 1 — Table S1. Summaries of the relevant findings from studies that have employed a HA‐tag for either the detection or purification of recombinant proteins expressed in the green microalgae Chlamydomonas reinhardtii. Table S2. Summary of studies that have employed a FLAG tag for either the detection or purification of recombinant proteins expressed in the green microalgae Chlamydomonas reinhardtii. Table S3. Summaries of the relevant findings from studies that have employed a Strep‐tag for either the detection or purification of recombinant proteins expressed in the green microalgae Chlamydomonas reinhardtii. Table S4. Summaries of the relevant findings from studies that have employed a His‐tag for either the detection or purification of recombinant proteins expressed in the green microalgae Chlamydomonas reinhardtii. Table S5. Summary of studies that have employed FPs for proving the genetic tractability of a microalgal species. Table S6. Summary of studies that have employed FPs for screening of expression levels mostly in the green microalga Chlamydomonas reinhardtii. Table S7. Summary of studies that have employed FPs for localisation studies mostly in the green microalga Chlamydomonas reinhardtii. Table S8. Summaries of studies that have employed ‘self‐cleaving’ 2A peptides in the expression of recombinant proteins in various microalgae species. [file TPJ-122-0-s002.docx]

# Supplementary Data

**Supplementary Table 1.** Summaries of the relevant findings from studies that have employed a H*A*-tag for either the detection or purification of recombinant proteins expressed in the green microalgae *Chlamydomonas reinhardtii*.

**Supplementary Table 2.** Summary of studies that have employed a FLAG-tag for either the detection or purification of recombinant proteins expressed in the green microalgae *Chlamydomonas reinhardtii*.

**Supplementary Table 3.** Summaries of the relevant findings from studies that have employed a Strep-tag for either the detection or purification of recombinant proteins expressed in the green microalgae *Chlamydomonas reinhardtii*.

**Supplementary Table 4.** Summaries of the relevant findings from studies that have employed a His-tag for either the detection or purification of recombinant proteins expressed in the green microalgae *Chlamydomonas* *reinhardtii*.

**Supplementary Table 5.** Summary of studies that have employed FPs for proving genetic tractability of a microalgal species.

**Supplementary Table 6.** Summary of studies that have employed FPs for screening of expression levels mostly in the green microalga *Chlamydomonas reinhardtii*.

**Supplementary Table 7.** Summary of studies that have employed FPs for localization studies mostly in the green microalga *Chlamydomonas reinhardtii*.

**Supplementary Table 8.** Summaries of studies that have employed "self-cleaving" 2A peptides in the expression of recombinant proteins in various microalgae species.

| Target protein | N- or C-terminal | Genome location | Relevant findings | Success or failure (✓/X) | Reference |
| --- | --- | --- | --- | --- | --- |
| FKBP12 | C-terminal | Nucleus | Succesful western blot with anti-HA antibody. Proper biofunctionality observed. | ✓ | (Crozet et al., 2018) |
| CodA, SplB and IBV-CTB | C-terminal | Chloroplast | Proteins detected via western blot using anti-HA antibody. | ✓ | (Larrea-Alvarez & Purton, 2020) |
| Bacteriocin LS2 | C-terminal | Nucleus | Successful western blot with anti-HA antibody. | ✓ | (Y. X. Liu et al., 2020) |
| AGG1 | C-terminal | Nucleus | Proteins detected by western blot using anti-HA antibody. | ✓ | (Ide et al., 2016) |
| Flagella proteins | C-terminal | Nucleus | Successful western blot with anti-HA antibody, purified by anti-HA resin and functionally characterised. | ✓ | (Lechtreck et al., 2009) |
| Ribosomal proteins | C-terminal | Chloroplast | Successful western blot with anti-HA antibody, purified by anti-HA resin and functionally characterised | ✓ | (Westrich et al., 2021) |
| Ycf3 & Ycf4 | C-terminal | Chloroplast | Successful western blot with anti-HA antibody, purified by anti-HA resin and functionally characterised. | ✓ | (Nellaepalli et al., 2018) |
| SARS-CoV-2 spike protein | C-terminal | Nucleus | Successful western blot using an anti-HA antibody, but purification using anti-HA resin was unsuccessful. | X | (Kiefer et al., 2022) |

**Supplementary Table 1.** Summaries of the relevant findings from studies that have employed a H*A*-tag for either the detection or purification of recombinant proteins expressed in the green microalgae *Chlamydomonas reinhardtii*. In the success or failure column, a “✓” denotes a success in using the specific tag, whereas a “X” denotes a relative failure.

| Target protein | N- or C-terminal tag | Genome location | Relevant findings | Success or failure (✓/X) | Reference |
| --- | --- | --- | --- | --- | --- |
| FKBP12 | N-terminal | Nucleus | Protein shown to be functional and then detected by western blot using anti-FLAG antibody. | ✓ | (Crozet et al., 2018) |
| Pfs48/45 | C-terminal | Chloroplast | Anti-FLAG resin used to purify the protein. | ✓ | (Jones et al., 2013) |
| hVEGF  & HMGB1 | C-terminal | Chloroplast | Proteins detected by western blot using anti-FLAG antibody, purified via anti-FLAG resin and then shown to be biofunctional. | ✓ | (Rasala et al., 2010) |
| Papillomavirus E7GGG | N-terminal | Chloroplast | FLAG-tag construct resulted in higher expression levels than His-tagged counterpart. | ✓ | (Demurtas et al., 2013) |
| ICAM-1 | C-terminal | Nucleus | Very high concentration of recombinant protein achieved via media optimisations, AEC and anti-flag resin purification. | ✓ | (Torres-Tiji et al., 2022) |

**Supplementary Table 2.** Summary of studies that have employed a FLAG-tag for either the detection or purification of recombinant proteins expressed in the green microalgae *Chlamydomonas reinhardtii*. In the success or failure column, a “✓” denotes a success in using the specific tag, whereas a “X” denotes a relative failure.

| Target protein | N- or C-terminal tag | Genome location | Relevant findings | Success or failure (✓/X) | Reference |
| --- | --- | --- | --- | --- | --- |
| ClpP complex | C-terminal | Chloroplast | Detection of protein using protein specific antibody after purification using Strep-Tactin affinity resin. | ✓ | (Derrien et al., 2012) |
| OCP2 | C-terminal | Nucleus | Detection of protein using protein specific antibody after purification using Strep-Tactin affinity resin. | ✓ | (Pivato et al., 2021) |
| FKBP12 | N-terminal | Nucleus | Strep-tag negatively impacted biofunctionality. | X | (Crozet et al., 2018) |
| hVEGF | C-terminal | Nucleus | Strep-tag negatively impacted biofunctionality in addition to transcript and protein accumulation. | X | (Jarquín-Cordero et al., 2020) |

**Supplementary Table 3.** Summaries of the relevant findings from studies that have employed a Strep-tag for either the detection or purification of recombinant proteins expressed in the green microalgae *Chlamydomonas reinhardtii*. In the success or failure column, a “✓” denotes a success in using the specific tag, whereas a “X” denotes a relative failure.

| Target protein | N- or C-terminal tag | Genome location | Relevant findings | Success or failure (✓/X) | Reference |
| --- | --- | --- | --- | --- | --- |
| 3 x mytichitin-A | C-terminal | Nucleus | Detection of protein via Coomassie blue staining after Ni-NTA purification and SEC. | ✓ | (Dong et al., 2018) |
| Human erythropoietin | C-terminal | Nucleus | Detection of protein by western blot using protein specific antibody after Ni^2+^-NTA IMAC. | ✓ | (Eichler-Stahlberg et al., 2009) |
| WSSV VP28 | C-terminal | Chloroplast | Detection of protein by western blot using protein specific antibody after Ni^2+^-NTA IMAC. | ✓ | (Kiataramgul et al., 2020) |
| ClpP complex | C-terminal | Chloroplast | Massive contamination of endogenous proteins using Ni^2+^-NTA IMAC. | X | (Derrien et al., 2012) |
| SARS-CoV-2 spike protein | C-terminal | Nucleus | Unsuccessful Ni^2+^-NTA IMAC. | X | (Kiefer et al., 2022) |
| FKBP12 | N-terminal | Nucleus | His-tag negatively impacted the protein’s biofunctionality. | X | (Crozet et al., 2018) |
| HPV 16 E7GGG | N-terminal | Chloroplast | His-tag resulted in low expression levels. | X | (Demurtas et al., 2013) |

**Supplementary Table 4.** Summaries of the relevant findings from studies that have employed a His-tag for either the detection or purification of recombinant proteins expressed in the green microalgae *Chlamydomonas* *reinhardtii*. In the success or failure column, a “✓” denotes a success in using the specific tag, whereas a “X” denotes a relative failure.

| **Tag** | **Target protein** | **N- or C-terminal tag** | **Transformed species** | **Genome location** | **Relevant findings** | **Success or failure** | **Reference** |
| --- | --- | --- | --- | --- | --- | --- | --- |
| GFP | only FP | none | *Tetradesmus obliquus* | nucleus | this species is transformable | ✓ | [Guo et al., 2013](https://doi.org/10.1016/j.jbiotec.2012.10.020) |
| sfGFP | only FP | none | *Cyanidioschyzon merolae* | nucleus | this species is transformable | ✓ | [Fujiwara et al., 2015](https://doi.org/10.3389/fpls.2015.00657) |
| Clover | only FP | none | *Neochloris oleoabundans* | nucleus | this species is transformable | ✓ | Munoz et al., 2019 |
| Clover | only FP | none | *Tetradesmus obliquus* | nucleus | this species is transformable | ✓ | [Munoz et al., 2019](https://doi.org/10.1016/j.algal.2019.101453) |
| mostly GFP | Mostly only FP | N/A | *many different marine protists* | nucleus/chloroplast | many species are transformable, some are not | ✓ | [Faktorova et al., 2020](https://www.nature.com/articles/s41592-020-0796-x) |

**Supplementary Table 5.** Summary of studies that have employed FPs for proving genetic tractability of a microalgal species. In the success or failure column, a “✓” denotes a success in using the specific FP.

| **Tag** | **Target protein** | **N- or C-terminal tag** | **Transformed species** | **Transformation location** | **Relevant findings** | **Success or failure** | **Reference** |
| --- | --- | --- | --- | --- | --- | --- | --- |
| mTagBFP | ble-2A-fluorescent protein | none | *Chlamydomonas reinhardtii* | nucleus | low signal-to-noise ratio due to background autofluorescence, 2-5 times of autofluorescence | ~ | [Rasala et al., 2013](https://onlinelibrary.wiley.com/doi/full/10.1111/tpj.12165) |
| mCerulean | ble-2A-fluorescent protein | none | *Chlamydomonas reinhardtii* | nucleus | low signal-to-noise ratio due to background autofluorescence, 2-5 times of autofluorescence, but good for localization study | ~ | [Rasala et al., 2013](https://onlinelibrary.wiley.com/doi/full/10.1111/tpj.12165) |
| mCerulean3 | terpene synthase | C | *Chlamydomonas reinhardtii* | nucleus | successful selection of highly expressing transformants | ✓ | [Einhaus et al., 2022](https://doi.org/10.1016/j.ymben.2022.06.002) |
| mCerulean3 | terpene synthase | C | *Chlamydomonas reinhardtii* | nucleus | successful selection of highly expressing transformants | ✓ | [Lauersen et al., 2018](https://www.sciencedirect.com/science/article/pii/S1096717618302064#f0010) |
| mCerulean3 | only FP | none | *Chlamydomonas reinhardtii* | nucleus | hard to visualise on culturing plates, hence difficult to screen for successful transformants | X | [Gutiérrez et al., 2022](https://www.frontiersin.org/journals/bioengineering-and-biotechnology/articles/10.3389/fbioe.2022.979607/full) |
| mTFP1 | only FP | none | *Chlamydomonas reinhardtii* | nucleus | easier distinguishable alternative to mCerulean that's also in the cyan region | ✓ | [Gutiérrez et al., 2022](https://www.frontiersin.org/journals/bioengineering-and-biotechnology/articles/10.3389/fbioe.2022.979607/full) |
| vivid Verde FP (VFP) | only FP | none | *Chlamydomonas reinhardtii* | chloroplast | functional as a reporter for expression optimisation, then replaced VFP with Cpl-1 and found that optimisation has to be done per target protein | ✓ | [Braun-Galleani et al., 2015](https://analyticalsciencejournals.onlinelibrary.wiley.com/doi/10.1002/biot.201400566) |
| GFP | Ble-GFP | C | *Chlamydomonas reinhardtii* | nucleus | characterisation of expression control via different riboswitches, nuclear target confirmed microscopically | ✓ | [Mehrshahi et al., 2020](https://doi.org/10.1021/acssynbio.0c00082) |
| GFP | only FP | none | *Chlamydomonas reinhardtii* | chloroplast | successful selection of highly expressing transformants for optimisation of light and trophic conditions | ✓ | [Carrera Pacheco et al., 2018](https://www.sciencedirect.com/science/article/pii/S2211926418300547) |
| GFP | ble-2A-fluorescent protein | none | *Chlamydomonas reinhardtii* | nucleus | low signal-to-noise ratio due to background autofluorescence, 2-5 times of autofluorescence | ✓ | [Rasala et al., 2013](https://onlinelibrary.wiley.com/doi/full/10.1111/tpj.12165) |
| mVenus | terpene synthase | C | *Chlamydomonas reinhardtii* | nucleus | successful selection of highly expressing transformants | ✓ | [Einhaus et al., 2021](https://pubs.acs.org/doi/10.1021/acssynbio.0c00632) |
| mVenus | terpene synthase | C | *Chlamydomonas reinhardtii* | nucleus | successful selection of highly expressing transformants | ✓ | [Einhaus et al., 2022](https://doi.org/10.1016/j.ymben.2022.06.002) |
| mVenus | terpene synthase | C | *Chlamydomonas reinhardtii* | nucleus | successful selection of highly expressing transformants | ✓ | [Lauersen et al., 2018](https://www.sciencedirect.com/science/article/pii/S1096717618302064#f0010) |
| Venus | ble-2A-fluorescent protein | none | *Chlamydomonas reinhardtii* | nucleus | high signal to-noise ratio of ~50 | ✓ | [Rasala et al., 2013](https://onlinelibrary.wiley.com/doi/full/10.1111/tpj.12165) |
| Venus | cpVenus | C | *Chlamydomonas reinhardtii* | nucleus | successful selection of highly expressing transformants for riboswitch characterisation | ✓ | [Mehrshahi et al., 2020](https://doi.org/10.1021/acssynbio.0c00082) |
| Venus | only FP | none | *Nannochloropsis oceanica* | nucleus | successful selection of highly expressing transformants, up to 4.9% of total protein was Venus | ✓ | [de Grahl et al., 2020](https://link.springer.com/article/10.1007/s00253-020-10789-4?) |
| LSSmOrange | only FP | none | *Chlamydomonas reinhardtii* | nucleus | long stokes shift, fluorescence readily detected on culturing plates | ✓ | [Gutiérrez et al., 2022](https://www.frontiersin.org/journals/bioengineering-and-biotechnology/articles/10.3389/fbioe.2022.979607/full) |
| tdTomato | ble-2A-fluorescent protein | none | *Chlamydomonas reinhardtii* | nucleus | very high signal to noise ratio of ~160, but tandem dimer so twice the size of other FPs +lower transformation efficiency | ✓ | [Rasala et al., 2013](https://onlinelibrary.wiley.com/doi/full/10.1111/tpj.12165) |
| mScarlet | only FP | none | *Chlamydomonas reinhardtii* | nucleus | fluorescence readily detected on culturing plates | ✓ | [Gutiérrez et al., 2022](https://www.frontiersin.org/journals/bioengineering-and-biotechnology/articles/10.3389/fbioe.2022.979607/full) |
| mRuby2 | terpene synthase | C | *Chlamydomonas reinhardtii* | nucleus | successful selection of highly expressing transformants | ✓ | [Lauersen et al., 2018](https://www.sciencedirect.com/science/article/pii/S1096717618302064#f0010) |
| mRuby2 | terpene synthase | C | *Chlamydomonas reinhardtii* | nucleus | successful selection of highly expressing transformants | ✓ | [Einhaus et al., 2022](https://doi.org/10.1016/j.ymben.2022.06.002) |
| LSSmCherry | only FP | none | *Chlamydomonas reinhardtii* | nucleus | long stokes shift, only very weak fluorescence signal on culturing plates | ~ | [Gutiérrez et al., 2022](https://www.frontiersin.org/journals/bioengineering-and-biotechnology/articles/10.3389/fbioe.2022.979607/full) |
| mCherry | ble-2A-fluorescent protein | none | *Chlamydomonas reinhardtii* | nucleus | high signal to-noise ratio of ~50 | ✓ | [Rasala et al., 2013](https://onlinelibrary.wiley.com/doi/full/10.1111/tpj.12165) |
| mCherry | Ble-2A-secretion peptide-mCherry | C | *Chlamydomonas reinhardtii* | nucleus | successful selection of highly expressing transformants for screening of multiple secretion peptides | ✓ | [Molino et al., 2018](https://doi.org/10.1371/journal.pone.0192433) |
| mCherry | Ble-2A-pJP30-mCherry | C | *Chlamydomonas reinhardtii* | nucleus | successful selection of highly expressing transformants for testing different nitrogen sources | ✓ | [Arias et al., 2024](https://onlinelibrary.wiley.com/doi/abs/10.1002/ceat.202300143) |
| mCherry | only fluorescent protein | none | *Chlamydomonas reinhardtii* | chloroplast | lower growth rate of mCherry expressing strain, authors suggest increased metbolic burden | ✓ | [Kim et al., 2020](https://link.springer.com/article/10.1007/s12033-020-00249-9) |
| mCherry | ble-E2A-mCherry | C | *Scenedesmus acutus* | nucleus | successful selection of highly expressing transformants | ✓ | [Suttangkakul et al., 2019](https://bmcbiotechnol.biomedcentral.com/articles/10.1186/s12896-018-0497-z) |
| LSSmKate2 | only fluorescent protein | none | *Chlamydomonas reinhardtii* | nucleus | long stokes shift, no fluorescence signal found on culturing plates | X | [Gutiérrez et al., 2022](https://www.frontiersin.org/journals/bioengineering-and-biotechnology/articles/10.3389/fbioe.2022.979607/full) |

**Supplementary Table 6.** Summary of studies that have employed FPs for screening of expression levels mostly in the green microalga *Chlamydomonas reinhardtii*. The entries are ordered by the wavelength of emission of the respective tag from blue to red. In the success or failure column, a “✓” denotes a success in using the specific tag, whereas a “X” denotes a relative failure, while “~” indicates uncertainty.

| **Tag** | **Target protein** | **N- or C-terminal tag** | **Transformed species** | **Transformation location** | **Relevant findings** | **Success or failure** | **Reference** |
| --- | --- | --- | --- | --- | --- | --- | --- |
| mCerulean | alpha-tubulin | N | *Chlamydomonas reinhardtii* | nucleus | functional and localised to the flagella and internal structures | ✓ | [Rasala et al., 2013](https://onlinelibrary.wiley.com/doi/full/10.1111/tpj.12165) |
| mCerulean3 | chloroplast targeting sequence CTP PSAD | C | *Chlamydomonas reinhardtii* | nucleus | functional and localised to chloroplast | ✓ | [Crozet et al., 2018](https://pubs.acs.org/doi/10.1021/acssynbio.8b00251) |
| mCerulean3 | chloroplast targeted terpene synthase | C | *Chlamydomonas reinhardtii* | nucleus | functional and localised to chloroplast | ✓ | [Lauersen et al., 2018](https://www.sciencedirect.com/science/article/pii/S1096717618302064#f0010) |
| GFP | only FP | none | *Chlamydomonas reinhardtii* | chloroplast | nuclear transformation of cTDA1 which enabled chloroplastic GFP expression via the atpA 5'UTR | ✓ | [Carrera-Pacheco et al., 2020](https://www.sciencedirect.com/science/article/pii/S2211926419309750) |
| Clover | only FP | none | *Chlamydomonas reinhardtii* | nucleus | functional and localised to cytosol | ✓ | Crozet et al., 2018 |
| mVenus | microbodies targeting peptide CrMSPTS1 (Malate Synthase PTS1-like sequence) | N | *Chlamydomonas reinhardtii* | nucleus | functional and localised to microbodies | ✓ | [Crozet et al., 2018](https://pubs.acs.org/doi/10.1021/acssynbio.8b00251) |
| mVenus | nuclear targeting peptide SV40 | N | *Chlamydomonas reinhardtii* | nucleus | functional and localised to nucleus | ✓ | [Crozet et al., 2018](https://pubs.acs.org/doi/10.1021/acssynbio.8b00251) |
| mVenus | PSR1 (Myb transcription factor: Phosphate Starvation Response 1) | C | *Chlamydomonas reinhardtii* | nucleus | functional and localised to nucleus | ✓ | [Slocombe et al., 2023](https://doi.org/10.3389/fpls.2023.1208168) |
| mVenus | terpene synthase | C | *Chlamydomonas reinhardtii* | nucleus | functional and localised to chloroplast | ✓ | [Lauersen et al., 2018](https://www.sciencedirect.com/science/article/pii/S1096717618302064#f0010) |
| mVenus | Cytochrome c6A | C | *Chlamydomonas reinhardtii* | nucleus | no fluorescent protein detected, tested with or without linkers. c6A is lumen localised which might cause difficulties with folding, transport etc. | X | Kosmützky, 2024 |
| YFP | targeting peptides RBCS2 (chloroplast), IFT46 (cilia), ATP2 (mitochondria) | C | *Chlamydomonas reinhardtii* | RNA-based transient | functional and localised to specific targeted compartment, transient RNA-based expression | ✓ | [Ye et al., 2024](https://doi.org/10.1016/j.nbt.2024.08.501) |
| YFP | NLS (nucleus) | N | *Chlamydomonas reinhardtii* | RNA-based transient | functional and localised to nucleus, transient RNA-based expression | ✓ | [Ye et al., 2024](https://doi.org/10.1016/j.nbt.2024.08.501) |
| mVenus | FKBP-rapamycin-binding FRB | N | *Cyanidioschyzon merolae* | nucleus | for protein knockdown via FRB mediated degradation, mVenus was functional in cytoplasm and broken down when FRB was present | ✓ | [Fujiwara et al., 2024](https://doi.org/10.1093/plphys/kiae316) |
| Venus | EPYC1 | C | *Chlamydomonas reinhardtii* | nucleus | functional and located to pyrenoid | ✓ | [Mackinder et al., 2016](https://www.pnas.org/doi/full/10.1073/pnas.1522866113) |
| Venus | 1034 different proteins | C | *Chlamydomonas reinhardtii* | nucleus | 1034 successfully localised of 3117 successfully cloned contrusts. whole atlas of localization data, with constructs available in the Chlamydomonas reinhardtii Library CLiP, constructs have Venus and tripleFLAG at C-terminus | ✓ | [Wang et al., 2023](https://www.sciencedirect.com/science/article/pii/S0092867423006761?via%3Dihub) |
| mRuby2 | mitochondria targeting peptide MTP ATPA | C | *Chlamydomonas reinhardtii* | nucleus | functional and localised to mitochondria | ✓ | [Crozet et al., 2018](https://pubs.acs.org/doi/10.1021/acssynbio.8b00251) |
| mRuby3 | various targeting peptides | C | *Phaeodactylum tricornutum* | nucleus | functional and localised to specific targeted compartment | ✓ | [Marter et a., 2020](https://doi.org/10.1016/j.protis.2020.125715) |
| mCherry | various targeting peptides | C | *Chlamydomonas reinhardtii* | nucleus | functional and localised to specific targeted compartment | ✓ | [Crozet et al., 2018](https://pubs.acs.org/doi/10.1021/acssynbio.8b00251) |
| mCherry | NLS (nucleus) | N | *Chlamydomonas reinhardtii* | RNA-based transient | functional and localised to nucleus, transient RNA-based expression | ✓ | [Ye et al., 2024](https://doi.org/10.1016/j.nbt.2024.08.501) |
| mCherry | RBCS1 | C | *Chlamydomonas reinhardtii* | nucleus | functional and located to pyrenoid | ✓ | [Mackinder et al., 2016](https://www.pnas.org/doi/full/10.1073/pnas.1522866113) |

**Supplementary table 7.** Summary of studies that have employed FPs for localization studies mostly in the green microalga Chlamydomonas reinhardtii. The entries are ordered by the wavelength of emission of the respective tag from blue to red. In the success or failure column, a “✓” denotes a success in using the specific tag, whereas a “X” denotes a relative failure, while “~” indicates uncertainty.

**Supplementary Table 8.** Summaries of studies that have employed "self-cleaving" 2A peptides in the expression of recombinant proteins in various microalgae species.

| Target Proteins | Microalgal species | 2A peptides analysed | Cleavage efficiency | Reference |
| --- | --- | --- | --- | --- |
| GFP and puromycin N-acetyltransferase | *Breviolum minutum* | P2A | Highly unreliable | (Gornik et al., 2022) |
| sfCherry and ShBle | Nannochloropsis salina | T2A, E2A, P2A and F2A | T2A (40%), E2A (37%), P2A (30%), F2A (21%) | (Koh et al., 2018) |
| GFP and ShBle | *C. reinhardtii* | F2A | Majority of protein was cleaved successfully | (Rasala et al., 2012) |
| hIL-2 and chloramphenicol acetyltransferase | *C. reinhardtii, C. vulgaris* and *Daniella salina* | Chimeric GSG-T2A-P2A | Total cleavage success | (Dehghani et al., 2020) |
