# Supplementary material for: A game of tag: A review of protein tags for the successful detection, purification and fluorescence labelling of proteins expressed in microalgae
Source: Plant J. 2025 Jun 19;122(6):e70272. doi: 10.1111/tpj.70272 (PMC12178573; doi:10.1111/tpj.70272)
Supplement: Supplementary file 2 — Supplementary Tables References [file TPJ-122-0-s001.docx]

**Supplementary Tables References**

Included here are all the references cited in the supplementary tables. Many references are also cited in the main text of the review.

Some references are reused in different tables, e.g. Crozet et al (2018), but for the purpose of clarity we have provided all of the references used within each table.

**Table S1**

Crozet, P., Navarro, F. J., Willmund, F., Mehrshahi, P., Bakowski, K., Lauersen, K. J., Pérez-Pérez, M. E., Auroy, P., Gorchs Rovira, A., Sauret-Gueto, S., Niemeyer, J., Spaniol, B., Theis, J., Trösch, R., Westrich, L. D., Vavitsas, K., Baier, T., Hübner, W., De Carpentier, F., … Lemaire, S. D. (2018). Birth of a Photosynthetic Chassis: A MoClo Toolkit Enabling Synthetic Biology in the Microalga *Chlamydomonas reinhardtii*. *ACS Synthetic Biology*, *7*(9), 2074–2086. https://doi.org/10.1021/acssynbio.8b00251

Ide, T., Mochiji, S., Ueki, N., Yamaguchi, K., Shigenobu, S., Hirono, M., & Wakabayashi, K. ichi. (2016). Identification of the *agg1* mutation responsible for negative phototaxis in a “wild-type” strain of *Chlamydomonas reinhardtii*. *Biochemistry and Biophysics Reports*, *7*, 379–385. https://doi.org/10.1016/j.bbrep.2016.07.016

Kiefer, A. M., Niemeyer, J., Probst, A., Erkel, G., & Schroda, M. (2022). Production and secretion of functional SARS-CoV-2 spike protein in *Chlamydomonas reinhardtii*. *Frontiers in Plant Science*, *13*(September), 1–12. https://doi.org/10.3389/fpls.2022.988870

Larrea-Alvarez, M., & Purton, S. (2020). Multigenic engineering of the chloroplast genome in the green alga *Chlamydomonas reinhardtii*. *Microbiology (United Kingdom)*, *166*(6), 510–515. https://doi.org/10.1099/mic.0.000910

Lechtreck, K. F., Luro, S., Awata, J., & Witman, G. B. (2009). HA-tagging of putative flagellar proteins in *Chlamydomonas reinhardtii* identifies a novel protein of intraflagellar transport complex B. *Cell Motility and the Cytoskeleton*, *66*(8), 469–482. <https://doi.org/10.1002/cm.20369>

Liu, Y. X., Li, Z. F., Lv, Y. J., Dong, B., & Fan, Z. C. (2020). *Chlamydomonas reinhardtii*-expressed multimer of Bacteriocin LS2 potently inhibits the growth of bacteria. *Process Biochemistry*, *95*(April), 139–147. https://doi.org/10.1016/j.procbio.2020.05.024

Nellaepalli, S., Ozawa, S.-I., Kuroda, H., & Takahashi, Y. (2018). The photosystem I assembly apparatus consisting of Ycf3–Y3IP1 and Ycf4 modules. *Nature Communications*, *9*(1), 2439. https://doi.org/10.1038/s41467-018-04823-3

Westrich, L. D., Gotsmann, V. L., Herkt, C., Ries, F., Kazek, T., Trösch, R., Armbruster, L., Mühlenbeck, J. S., Ramundo, S., Nickelsen, J., Finkemeier, I., Wirtz, M., Storchová, Z., Räschle, M., & Willmund, F. (2021). The versatile interactome of chloroplast ribosomes revealed by affinity purification mass spectrometry. *Nucleic Acids Research*, *49*(1), 400–415. <https://doi.org/10.1093/nar/gkaa1192>

**Table S2**

Crozet, P., Navarro, F. J., Willmund, F., Mehrshahi, P., Bakowski, K., Lauersen, K. J., Pérez-Pérez, M. E., Auroy, P., Gorchs Rovira, A., Sauret-Gueto, S., Niemeyer, J., Spaniol, B., Theis, J., Trösch, R., Westrich, L. D., Vavitsas, K., Baier, T., Hübner, W., De Carpentier, F., … Lemaire, S. D. (2018). Birth of a Photosynthetic Chassis: A MoClo Toolkit Enabling Synthetic Biology in the Microalga *Chlamydomonas reinhardtii*. *ACS Synthetic Biology*, *7*(9), 2074–2086. https://doi.org/10.1021/acssynbio.8b00251

Demurtas, O. C., Massa, S., Ferrante, P., Venuti, A., Franconi, R., & Giuliano, G. (2013). A *Chlamydomonas*-Derived Human Papillomavirus 16 E7 Vaccine Induces Specific Tumor Protection. *PLoS ONE*, *8*(4), 1–9. https://doi.org/10.1371/journal.pone.0061473

Jones, C. S., Luong, T., Hannon, M., Tran, M., Gregory, J. A., Shen, Z., Briggs, S. P., & Mayfield, S. P. (2013). Heterologous expression of the C-terminal antigenic domain of the malaria vaccine candidate Pfs48/45 in the green algae *Chlamydomonas reinhardtii*. *Applied Microbiology and Biotechnology*, *97*(5), 1987–1995. <https://doi.org/10.1007/s00253-012-4071-7>

Rasala, B. A., Muto, M., Lee, P. A., Jager, M., Cardoso, R. M. F., Behnke, C. A., Kirk, P., Hokanson, C. A., Crea, R., Mendez, M., & Mayfield, S. P. (2010). Production of therapeutic proteins in algae, analysis of expression of seven human proteins in the chloroplast of *Chlamydomonas reinhardtii*. *Plant Biotechnology Journal*, *8*(6), 719–733. https://doi.org/10.1111/j.1467-7652.2010.00503.x

Torres-Tiji, Y., Fields, F. J., Yang, Y., Heredia, V., Horn, S. J., Keremane, S. R., Jin, M. M., & Mayfield, S. P. (2022). Optimized production of a bioactive human recombinant protein from the microalgae *Chlamydomonas reinhardtii* grown at high density in a fed-batch bioreactor. *Algal Research*, *66*(July), 102786. https://doi.org/10.1016/j.algal.2022.102786

**Table S3**

Crozet, P., Navarro, F. J., Willmund, F., Mehrshahi, P., Bakowski, K., Lauersen, K. J., Pérez-Pérez, M. E., Auroy, P., Gorchs Rovira, A., Sauret-Gueto, S., Niemeyer, J., Spaniol, B., Theis, J., Trösch, R., Westrich, L. D., Vavitsas, K., Baier, T., Hübner, W., De Carpentier, F., … Lemaire, S. D. (2018). Birth of a Photosynthetic Chassis: A MoClo Toolkit Enabling Synthetic Biology in the Microalga *Chlamydomonas reinhardtii*. *ACS Synthetic Biology*, *7*(9), 2074–2086. https://doi.org/10.1021/acssynbio.8b00251

Derrien, B., Majeran, W., Effantin, G., Ebenezer, J., Friso, G., van Wijk, K. J., Steven, A. C., Maurizi, M. R., & Vallon, O. (2012). The purification of the *Chlamydomonas reinhardtii* chloroplast ClpP complex: Additional subunits and structural features. *Plant Molecular Biology*, *80*(2), 189–202. https://doi.org/10.1007/s11103-012-9939-5

Jarquín-Cordero, M., Chávez, M. N., Centeno-Cerdas, C., Bohne, A. V., Hopfner, U., Machens, H. G., Egaña, J. T., & Nickelsen, J. (2020). Towards a biotechnological platform for the production of human pro-angiogenic growth factors in the green alga *Chlamydomonas reinhardtii*. *Applied Microbiology and Biotechnology*, *104*(2), 725–739. https://doi.org/10.1007/s00253-019-10267-6

Pivato, M., Perozeni, F., Licausi, F., Cazzaniga, S., & Ballottari, M. (2021). Heterologous expression of cyanobacterial Orange Carotenoid Protein (OCP2) as a soluble carrier of ketocarotenoids in *Chlamydomonas reinhardtii*. *Algal Research*, *55*, 102255. https://doi.org/10.1016/j.algal.2021.102255

**Table S4**

Crozet, P., Navarro, F. J., Willmund, F., Mehrshahi, P., Bakowski, K., Lauersen, K. J., Pérez-Pérez, M. E., Auroy, P., Gorchs Rovira, A., Sauret-Gueto, S., Niemeyer, J., Spaniol, B., Theis, J., Trösch, R., Westrich, L. D., Vavitsas, K., Baier, T., Hübner, W., De Carpentier, F., … Lemaire, S. D. (2018). Birth of a Photosynthetic Chassis: A MoClo Toolkit Enabling Synthetic Biology in the Microalga *Chlamydomonas reinhardtii*. *ACS Synthetic Biology*, *7*(9), 2074–2086. https://doi.org/10.1021/acssynbio.8b00251

Demurtas, O. C., Massa, S., Ferrante, P., Venuti, A., Franconi, R., & Giuliano, G. (2013). A *Chlamydomonas*-Derived Human Papillomavirus 16 E7 Vaccine Induces Specific Tumor Protection. *PLoS ONE*, *8*(4), 1–9. https://doi.org/10.1371/journal.pone.0061473

Derrien, B., Majeran, W., Effantin, G., Ebenezer, J., Friso, G., van Wijk, K. J., Steven, A. C., Maurizi, M. R., & Vallon, O. (2012). The purification of the *Chlamydomonas reinhardtii* chloroplast ClpP complex: Additional subunits and structural features. *Plant Molecular Biology*, *80*(2), 189–202. https://doi.org/10.1007/s11103-012-9939-5

Dong, B., Cheng, R. Q., Liu, Q. Y., Wang, J., & Fan, Z. C. (2018). Multimer of the antimicrobial peptide Mytichitin-A expressed in *Chlamydomonas reinhardtii* exerts a broader antibacterial spectrum and increased potency. *Journal of Bioscience and Bioengineering*, *125*(2), 175–179. https://doi.org/10.1016/j.jbiosc.2017.08.021

Eichler-Stahlberg, A., Weisheit, W., Ruecker, O., & Heitzer, M. (2009). Strategies to facilitate transgene expression in *Chlamydomonas reinhardtii*. *Planta*, *229*(4), 873–883. https://doi.org/10.1007/s00425-008-0879-x

Kiataramgul, A., Maneenin, S., Purton, S., Areechon, N., Hirono, I., Brocklehurst, T. W., & Unajak, S. (2020). An oral delivery system for controlling white spot syndrome virus infection in shrimp using transgenic microalgae. *Aquaculture*, *521*(January), 735022. https://doi.org/10.1016/j.aquaculture.2020.735022

Kiefer, A. M., Niemeyer, J., Probst, A., Erkel, G., & Schroda, M. (2022). Production and secretion of functional SARS-CoV-2 spike protein in *Chlamydomonas reinhardtii*. *Frontiers in Plant Science*, *13*(September), 1–12. https://doi.org/10.3389/fpls.2022.988870

**Table S5**

Faktorová, D., Nisbet, R. E. R., Fernández Robledo, J. A., Casacuberta, E., Sudek, L., Allen, A. E., Ares, M., Aresté, C., Balestreri, C., Barbrook, A. C., Beardslee, P., Bender, S., Booth, D. S., Bouget, F. Y., Bowler, C., Breglia, S. A., Brownlee, C., Burger, G., Cerutti, H., … Lukeš, J. (2020). Genetic tool development in marine protists: emerging model organisms for experimental cell biology. *Nature Methods*, *17*(5), 481–494. https://doi.org/10.1038/s41592-020-0796-x

Fujiwara, T., Kanesaki, Y., Hirooka, S., Era, A., Sumiya, N., Yoshikawa, H., Tanaka, K., & Miyagishima, S. Y. (2015). A nitrogen source-dependent inducible and repressible gene expression system in the red alga Cyanidioschyzon merolae. *Frontiers in Plant Science*, *6*(AUG), 1–10. https://doi.org/10.3389/fpls.2015.00657

Guo, S. L., Zhao, X. Q., Tang, Y., Wan, C., Alam, M. A., Ho, S. H., Bai, F. W., & Chang, J. S. (2013). Establishment of an efficient genetic transformation system in Scenedesmus obliquus. *Journal of Biotechnology*, *163*(1), 61–68. <https://doi.org/10.1016/j.jbiotec.2012.10.02>

Muñoz, C. F., Sturme, M. H. J., D’Adamo, S., Weusthuis, R. A., & Wijffels, R. H. (2019). Stable transformation of the green algae Acutodesmus obliquus and Neochloris oleoabundans based on E. coli conjugation. *Algal Research*, *39*(December 2018). <https://doi.org/10.1016/j.algal.2019.101453>

**Table S6**

Arias, C. A. D., Matsudo, M. C., Ferreira-Camargo, L. S., Molino, J. V. D., Mayfield, S. P. and de Carvalho, J. C. M. (2024). Effect of Macronutrients on Recombinant mCherry Production by Microalga. *Chem. Eng. Technol.* 1–10. <https://doi.org/10.1002/ceat.202300143>

Braun-Galleani, S., Baganz, F. and Purton, S. (2015). Improving recombinant protein production in the *Chlamydomonas reinhardtii* chloroplast using vivid Verde Fluorescent Protein as a reporter. *Biotechnol. J.* 10, 1289–1297. https://doi.org/10.1002/biot.201400566

Carrera Pacheco, S. E., Hankamer, B. and Oey, M. (2018). Optimising light conditions increases recombinant protein production in *Chlamydomonas reinhardtii* chloroplasts. *Algal Res.* 32, 329–340. https://doi.org/10.1016/j.algal.2018.04.011

de Grahl, I., Rout, S. S., Maple-Grødem, J. and Reumann, S. (2020). Development of a constitutive and an auto-inducible high-yield expression system for recombinant protein production in the microalga Nannochloropsis oceanica. *Appl. Microbiol. Biotechnol.* 104, 8747–8760. https://doi.org/10.1007/s00253-020-10789-4

Einhaus, A., Baier, T., Rosenstengel, M., Freudenberg, R. A., & Kruse, O. (2021). Rational Promoter Engineering Enables Robust Terpene Production in Microalgae. *ACS Synthetic Biology*, *10*(4), 847–856. https://doi.org/10.1021/acssynbio.0c00632

Einhaus, A., Steube, J., Freudenberg, R. A., Barczyk, J., Baier, T. and Kruse, O. (2022). Engineering a powerful green cell factory for robust photoautotrophic diterpenoid production. *Metabolic Engineering* 73, 82–90. <https://doi.org/10.1016/j.ymben.2022.06.002>

Gutiérrez, S., Wellman, G. B., & Lauersen, K. J. (2022). Teaching an old ‘doc’ new tricks for algal biotechnology: Strategic filter use enables multi-scale fluorescent protein signal detection. Frontiers in Bioengineering and Biotechnology, 10. https://doi.org/10.3389/fbioe.2022.979607

Kim, S. Y., Kim, K. W., Kwon, Y. M. and Kim, J. Y. H. (2020). mCherry Protein as an In Vivo Quantitative Reporter of Gene Expression in the Chloroplast of *Chlamydomonas reinhardtii*. *Mol. Biotechnol.* 62, 297–305. https://doi.org/10.1007/s12033-020-00249-9

Lauersen, K. J., Wichmann, J., Baier, T., Kampranis, S. C., Pateraki, I., Møller, B. L., & Kruse, O. (2018). Phototrophic production of heterologous diterpenoids and a hydroxy-functionalized derivative from *Chlamydomonas reinhardtii*. *Metabolic Engineering*, *49*(May), 116–127. https://doi.org/10.1016/j.ymben.2018.07.005

Mehrshahi, P., Nguyen, G. T. D. T., Gorchs Rovira, A., Sayer, A., Llavero-Pasquina, M., Lim Huei Sin, M., Medcalf, E. J., Mendoza-Ochoa, G. I., Scaife, M. A., & Smith, A. G. (2020). Development of Novel Riboswitches for Synthetic Biology in the Green Alga *Chlamydomonas*. *ACS Synthetic Biology*, *9*(6), 1406–1417. https://doi.org/10.1021/acssynbio.0c00082

Molino, J. V. D., de Carvalho, J. C. M. and Mayfield, S. P. (2018). Comparison of secretory signal peptides for heterologous protein expression in microalgae: Expanding the secretion portfolio for *Chlamydomonas reinhardtii*. *PLoS One* 13, 1–20. https://doi.org/10.1371/journal.pone.0192433

Rasala, B. A., Barrera, D. J., Ng, J., Plucinak, T. M., Rosenberg, J. N., Weeks, D. P., Oyler, G. A., Peterson, T. C., Haerizadeh, F., & Mayfield, S. P. (2013). Expanding the spectral palette of fluorescent proteins for the green microalga *Chlamydomonas reinhardtii*. *Plant Journal*, *74*(4), 545–556. https://doi.org/10.1111/tpj.12165

Suttangkakul, A., Sirikhachornkit, A., Juntawong, P., Puangtame, W., Chomtong, T., Srifa, S., Sathitnaitham, S., Dumrongthawatchai, W., Jariyachawalid, K. and Vuttipongchaikij, S. (2019). Evaluation of strategies for improving the transgene expression in an oleaginous microalga Scenedesmus acutus. *BMC Biotechnol.* 19, 1–15. <https://doi.org/1010.1186/s12896-018-0497-z>

**Table S7**

Carrera Pacheco, S. E., Hankamer, B. and Oey, M. (2018). Optimising light conditions increases recombinant protein production in *Chlamydomonas reinhardtii* chloroplasts. *Algal Res.* 32, 329–340. https://doi.org/10.1016/j.algal.2018.04.011

Crozet, P., Navarro, F. J., Willmund, F., Mehrshahi, P., Bakowski, K., Lauersen, K. J., Pérez-Pérez, M. E., Auroy, P., Gorchs Rovira, A., Sauret-Gueto, S., Niemeyer, J., Spaniol, B., Theis, J., Trösch, R., Westrich, L. D., Vavitsas, K., Baier, T., Hübner, W., De Carpentier, F., … Lemaire, S. D. (2018). Birth of a Photosynthetic Chassis: A MoClo Toolkit Enabling Synthetic Biology in the Microalga *Chlamydomonas reinhardtii*. *ACS Synthetic Biology*, *7*(9), 2074–2086. https://doi.org/10.1021/acssynbio.8b00251

Fujiwara, T., Hirooka, S., Yamashita, S., Yagisawa, F. and Miyagishima, S. Y. (2024). Development of a rapamycin-inducible protein-knockdown system in the unicellular red alga Cyanidioschyzon merolae. *Plant Physiol.* 196, 77–94. https://doi.org/10.1093/plphys/kiae316

Kosmützky, D. G. (2024). Exploring the Function of Cytochrome *c*_6A_.PhD thesis, University of Cambridge.

Lauersen, K. J., Wichmann, J., Baier, T., Kampranis, S. C., Pateraki, I., Møller, B. L., & Kruse, O. (2018). Phototrophic production of heterologous diterpenoids and a hydroxy-functionalized derivative from *Chlamydomonas reinhardtii*. *Metabolic Engineering*, *49*(May), 116–127. https://doi.org/10.1016/j.ymben.2018.07.005

Mackinder, L. C. M., Meyer, M. T., Mettler-Altmann, T., Chen, V. K., Mitchell, M. C., Caspari, O., Rosenzweig, E. S. F., Pallesen, L., Reeves, G., Itakura, A., et al. (2016). A repeat protein links Rubisco to form the eukaryotic carbon-concentrating organelle. *Proc. Natl. Acad. Sci. U. S. A.* 113, 5958–5963. https://doi.org/10.1073/pnas.1522866113

Marter, P., Schmidt, S., Kiontke, S. and Moog, D. (2020). Optimized mRuby3 is a Suitable Fluorescent Protein for in vivo Co-localization Studies with GFP in the Diatom Phaeodactylum tricornutum. *Protist* 171, 125715. https://doi.org/10.1016/j.protis.2020.125715

Rasala, B. A., Barrera, D. J., Ng, J., Plucinak, T. M., Rosenberg, J. N., Weeks, D. P., Oyler, G. A., Peterson, T. C., Haerizadeh, F., & Mayfield, S. P. (2013). Expanding the spectral palette of fluorescent proteins for the green microalga *Chlamydomonas reinhardtii*. *Plant Journal*, *74*(4), 545–556. https://doi.org/10.1111/tpj.12165

Slocombe, S. P., Zúñiga-Burgos, T., Chu, L., Mehrshahi, P., Davey, M. P., Smith, A. G., Camargo-Valero, M. A. and Baker, A. (2023). Overexpression of PSR1 in *Chlamydomonas reinhardtii* induces luxury phosphorus uptake. *Front. Plant Sci.* 14, 1–21. https://doi.org/10.3389/fpls.2023.1208168

Wang, L., Patena, W., Van Baalen, K. A., Xie, Y., Singer, E. R., Gavrilenko, S., Warren-Williams, M., Han, L., Harrigan, H. R., Hartz, L. D., Chen, V., Ton, V. T. N. P., Kyin, S., Shwe, H. H., Cahn, M. H., Wilson, A. T., Onishi, M., Hu, J., Schnell, D. J., … Jonikas, M. C. (2023). A chloroplast protein atlas reveals punctate structures and spatial organization of biosynthetic pathways. *Cell*, *186*(16), 3499-3518.e14. <https://doi.org/10.1016/j.cell.2023.06.008>

Ye, L., Liao, T., Deng, X., Long, H., Liu, G., Ke, W., & Huang, K. (2024). Establishment of an RNA-based transient expression system in the green alga *Chlamydomonas reinhardtii*. *New Biotechnology*, *83*(August), 175–187. https://doi.org/10.1016/j.nbt.2024.08.501

**Table S8**

Dehghani, J., Adibkia, K., Movafeghi, A., Pourseif, M. M., & Omidi, Y. (2020). Designing a new generation of expression toolkits for engineering of green microalgae; robust production of human interleukin-2. *BioImpacts*, *10*(4), 259–268. https://doi.org/10.34172/bi.2020.33

Gornik, S. G., Maegele, I., Hambleton, E. A., Voss, P. A., Waller, R. F., & Guse, A. (2022). Nuclear transformation of a dinoflagellate symbiont of corals. *Frontiers in Marine Science*, *9*(November), 1–15. https://doi.org/10.3389/fmars.2022.1035413

Koh, H. G., Kang, N. K., Kim, E. K., Jeon, S., Shin, S. E., Lee, B., & Chang, Y. K. (2018). Advanced multigene expression system for Nannochloropsis salina using 2A self-cleaving peptides. *Journal of Biotechnology*, *278*(November 2017), 39–47. <https://doi.org/10.1016/j.jbiotec.2018.04.017>

Rasala, B. A., Lee, P. A., Shen, Z., Briggs, S. P., Mendez, M., & Mayfield, S. P. (2012). Robust expression and secretion of xylanase1 in *Chlamydomonas reinhardtii* by fusion to a selection gene and processing with the FMDV 2A peptide. *PLoS ONE*, *7*(8). https://doi.org/10.1371/journal.pone.0043349
